# Magnitude and factors associated with intimate partner violence against pregnant women in Ethiopia: a multilevel analysis of 2016 Ethiopian demographic and health survey

**DOI:** 10.1186/s12889-022-12720-0

**Published:** 2022-02-11

**Authors:** Alemneh Mekuriaw Liyew, Adugnaw Zeleke Alem, Hiwotie Getaneh Ayalew

**Affiliations:** 1 Department of Epidemiology and Biostatistics, Institute of Public Health, College of Medicine and Health Sciences and Comprehensive Specialized Hospital, University of Gondar, Gondar, Ethiopia; 2 Department of midwifery, School of Nursing and Midwifery, College of Medicine and Health Sciences, Wollo University, Dessie, Ethiopia

**Keywords:** Magnitude, Intimate partner violence pregnancy, Multilevel analysis, Ethiopia

## Abstract

**Background:**

Intimate partner violence (IPV) is defined as acts of physical aggression, sexual coercion, psychological/emotional abuse, or controlling behaviors by a current or former partner or spouse. IPV has a special concern for pregnant women since it leads to higher rates of miscarriage, several complications including adverse birth outcomes. So far, the effect of contextual factors on IPV was largely overlooked. Therefore, this study aimed to assess the magnitude and factors associated with IPV among pregnant women in Ethiopia.

**Method:**

Data from the 2016 Ethiopian Demographic and Health Survey was used for this study. A total of 4167 (weighted sample) pregnant women were included in the analysis. The multi-level logistic regression model was fitted to identify factors associated with IPV. Finally, the adjusted odds ratio (AOR) with 95% CI and random effects for the multilevel logistic regression model was reported.

**Results:**

In this study, the overall magnitude of IPV among pregnant women was 28.74 (95% CI 27.38, 30.13) with emotional violence being the most common (24.09%) type. In the multi-level analysis, women with no education (AOR = 2.07; 95%CI 1.23, 3.48), primary education (AOR = 2.04; 95%CI:1.24, 3.38), and secondary education (AOR = 1.53; 95%CI:1.29.2.62), women from households with poorest (AOR = 1.72; 95%CI: 1.16, 2.56), poorer (AOR = 1.62;95% CI:1.09, 2.41), middle (AOR = 1.74;95%CI:1.17, 2.56), and richer (AOR = 1.58;95%CI: 1.08, 2.33) wealth index, women aged 35–39 years (AOR = 1.28;95%CI:1.01, 1.63) and 40–49 years (AOR = 1.78;95%CI:1.28, 2.45) and those from pastoral (AOR = 1.47;95%CI:1.04, 1.93) and agrarian regions (AOR = 1.32;95%CI 1.02, 1.88) had a higher likelihood of having IPV. Of the partner-related factors, women with husbands who drink alcohol (AOR = 2.94; 95%CI: 2.36, 3.42) and secondary educational level (AOR = 1.47; 95%CI 1.02, 2.12) had higher odds of experiencing IPV during pregnancy.

**Conclusion:**

Intimate partner violence during pregnancy is a public health problem in Ethiopia. Therefore, improving the educational status of women and their husbands, improving the economic capacity of women, and promoting the healthy behavior of husbands by reducing the alcohol consumption in those agrarian and pastoral regions of Ethiopia is vital to reduce the magnitude of IPV.

## Background

Intimate partner violence (IPV) which is defined as acts of physical aggression, sexual coercion, emotional abuse by a current or former partner or spouse [1] against women is the commonest source of violence directed to women [2]. It has three forms such as physical, sexual, and emotional violence [3].

In low-income countries, reported intimate partner violence (IPV) varied widely from less than 5% in Armenia and Comoros to more than 40% in Afghanistan with richer and more empowered women having less IPV [4]. Studies conducted in sub-Saharan African and Asian countries showed an IPV rate ranging from 28% in Madagascar, 74% in Ethiopia, and 57% in India to 87% in Jordan [5]. The evidence from a multi-country study indicated an IPV rate ranging from 18.5 to 75.8%. In sub-Saharan Africa, it is verified that the magnitude of intimate partner violence is higher than non-intimate partner violence [6, 7].

The magnitude of IPV among pregnant women was 33 and 37% in Nigeria [8] and Kenya [9] respectively. In Ethiopia, it varies across different parts of the country ranging from 24.5% in the northwest and southeast [10, 11], 37.5% in the north [12], to 39.81% in the eastern [13] part of Ethiopia.

Intimate partner violence is considered a global problem with serious public health and human rights implications. It affects all the spheres of women’s lives such as self-esteem, productivity, autonomy, capacity to care for themselves and their children, and ability to participate in social activities. Besides, it directly or indirectly leads to serious injury, disability mental disorders, substance use, and even death [6, 14].

Especially for pregnant women, IPV has special concern due to the potential negative impacts for both themselves and their fetuses. It may lead to higher rates of miscarriage, many complications (such as abruption placenta, placenta previa, preeclampsia, gestational diabetes antepartum hemorrhage e.t.c), sexually transmitted infections, and a higher prevalence of mental disorders (such as depression, anxiety, sleep disorders, and eating disorders) [15–18].

Furthermore, IPV during pregnancy is related with high perinatal and neonatal mortality [19]. Intrauterine growth retardation, preterm delivery, and low birth weight are common neonatal complications which happen as result of pregnancy related violence [20–23]. Moreover, increased intensity and frequency of pregnancy related violence was reported among women who were victims of violence just before and during pregnancy [24]. Another study found that pregnant abused women had more severe injuries than did nonpregnant abused women [25].

In the previous studies, partner alcohol consumption [9, 26, 27] husband education [9, 27–29], women education [27, 28, 30], age of women [31, 32], women decision-making capacity [33], history of IPV [32], place of residence [27] and household resources [34] are significantly associated with intimate partner violence.

Though different studies [10–13] were conducted in Ethiopia, all of them focus on the specific part of Ethiopia and assess the effect of individual-level factors without considering the context of the community where the women are dwelling and others focus on IPV among reproductive-age women [35, 36].

Although the effect of IPV is superior among pregnant women, there is limited evidence on the magnitude and associated factors of IPV among pregnant women in Ethiopia. Knowing the prevalence of intimate partner violence during pregnancy is the first step for the development and implementation of interventions to prevent and treat sequelae. Therefore, the current study aimed to assess the magnitude and factors associated with IPV among pregnant women in Ethiopia.

## Method

### Data source and setting

This study used Demographic and Health Survey (DHS) data which were collected using a cross-sectional study design. The DHS collects a wide range of objective and self-reported data with a strong focus on indicators of fertility, reproductive health, maternal and child health, mortality, nutrition, and self-reported health behaviors among adults. Data from DHS facilitate epidemiological research focused on monitoring prevalence, trends, and inequalities. It drew nationally representative samples for the country’s population. A detailed description of the nature of demographic and health survey datasets was published elsewhere [37]. The Ethiopian demographic and health survey is part of the worldwide DHS project. Therefore, the current study was based on data from the fourth Ethiopian demographic and health survey which was conducted in 2016.

### Sample size and sampling procedure

**Fig. 1:**
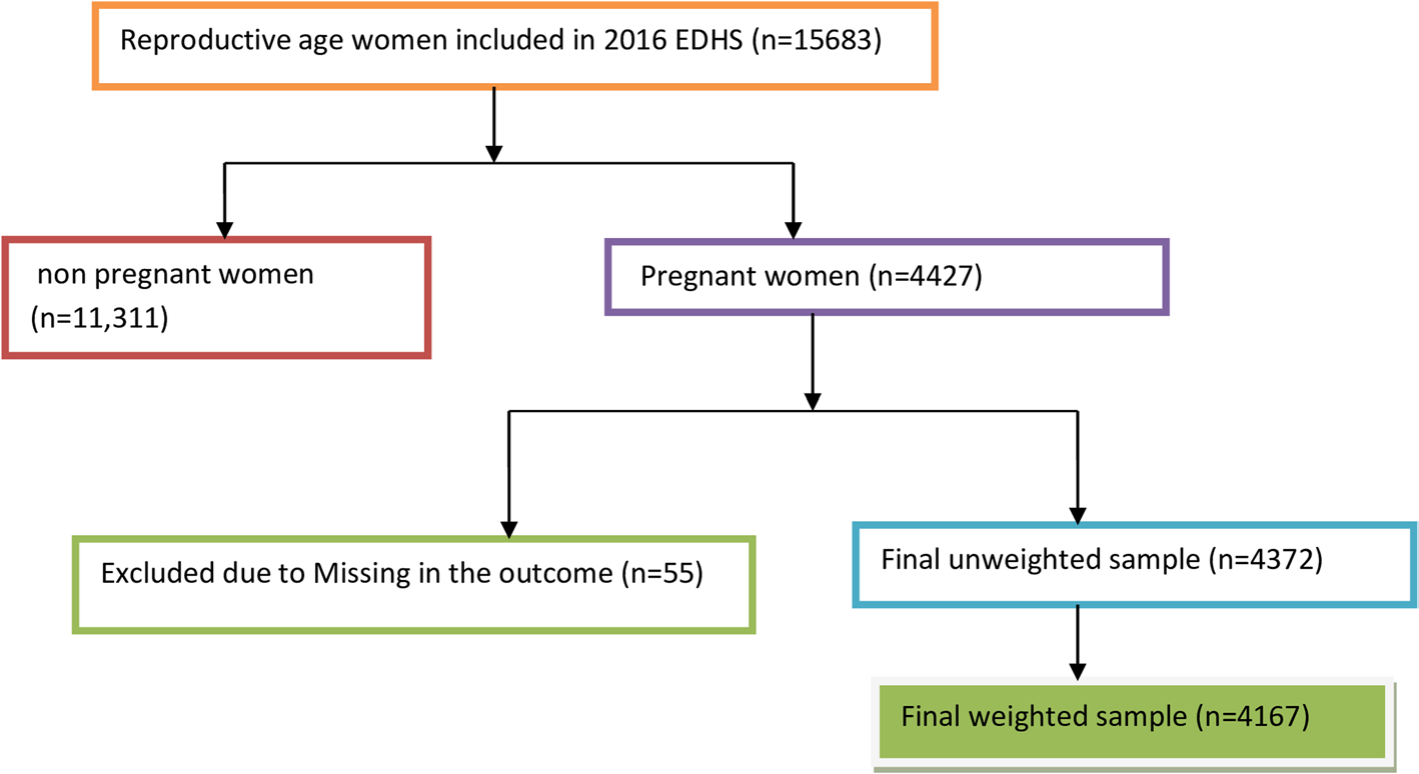
The data extraction procedure and sample size

To assure national representativeness, the 2016 Ethiopian demographic and health survey (EDHS) employs a stratified two-stage cluster sampling technique. In the first stage, a total of 645 enumeration areas (EAs) that represent the entire country were randomly selected from the sampling frame (i.e. developed from the 2007 census). The second stage is the systematic sampling of households listed in each cluster or EA and interviews are conducted in selected households with target populations (women aged 15–49 and men aged 15–64). A full description of EDHS is published elsewhere [38]. A total of 15,683 reproductive-age women were included in EDHS 2016. Of these, only pregnant women (pregnant at the time of data collection, give birth within the last five years, or had a history of terminated pregnancy) were included in the current study to assess whether they have experienced intimate partner violence during any of their pregnancy. So, the study participants were women aged 15–49 who were selected and interviewed for the domestic violence module and who have ever been pregnant. The sample is extracted as (v044 = 1; selected for domestic violence module) and (v201 > 0: woman had given live birth) or v213 = 1; pregnant at the time of interview) or v228 = 1(woman had history of terminated pregnancy). Therefore, after excluding the missing values the final weighted sample size was 4167 with 640 clusters (EA) (Fig. 1).

### Dependent variable

The modified Conflict Tactic Scales of Straus [39] was used to measure intimate partner violence (IPV). Women were asked whether or not they had experienced the acts forwarded by their husband/partner for currently married women and recent husband/partner for previously married women during any of her pregnancies. Then, the women’s self-reported responses to questions were used to decide the women’s IPV experience (Table 1). Thus, respondents were categorized as having experienced IPV if they reported experiencing at least one act of IPV during pregnancy.

**Table 1:** Questions used to assess intimate partner violence

Types of IPV	Questions used to assess violence
Physical violence	Ever been kicked or dragged by your husband?
	Ever been strangled or burned by a husband?
	Ever been threatened with a knife, gun, or another weapon?
Sexual violence	Ever been physically forced to have unwanted sex by your husband?
	Ever been forced to do other sexual acts by your husband?
	Ever been forced to perform sexual acts respondent didn’t want to?
Emotional violence	Ever been humiliated by your husband?
	Ever been threatened with harm by your husband?
	Ever been insulted or made to feel bad by your husband?

### Independent variables

Educational status of women (no formal education, primary, secondary, higher education), age of women [4, 15–49], maternal occupation (working, not working), husband education (no formal education, primary, secondary, higher education), husband occupation (not working, working), husband drinking alcohol (yes, no), wealth status (poorest, poorer, middle, richer and richest), media exposure (yes, no), sex of household head (male, female) were considered as individual-level variables.

Of the community level variables, place of residence (urban, rural), and region (city dwellers, pastoral, agrarian), were directly accessible from EDHS dataset. However, community poverty level (low, high), community illiteracy level (low, high), and community media exposure (low, high) were aggregated from individual-level characteristics at the cluster level. The distribution of the proportion values computed for each community was checked by using the histogram. Therefore, for dichotomization, mean and median values were used for normaly distributed and skewed proportions respectively.

### Multilevel binary logistic regression analysis

In large surveys, the clustering sampling approach commonly leads to correlation among the observations. This dependence among observations often comes from several levels of the hierarchy due to the multi-stage sampling scheme employed. In this case, applying traditional single-level statistical models leads to biased parameter estimation [40]. Therefore, to draw appropriate inferences and conclusions from hierarchical large survey data, we applied multilevel modeling techniques. Since the outcome variable is dichotomous, multilevel binary logistic regression analysis is employed for the current study.

Thus, the Intraclass Correlation Coefficient (ICC), and Proportional Change in Variance (PCV), were conducted to assess the significance of variability in experiencing IPV across the communities [41]. The proportional change in variance is calculated as;

$$\mathrm{PCV}=\left[\left(\mathrm{VA}-\mathrm{VB}\right)/\mathrm{VA}\right]\ast 100$$

Where; where VA = variance of the initial model, and VB = variance of the model with more terms.

A total of four models were fitted; the null model (with no predictors), model II (adjusted for individual-level variables only), model III (adjusted for community-level variables only), and model IV (model adjustment for both individual and community-level variables simultaneously) were fitted. The deviance was used for model comparison. Finally adjusted odds ratio (AOR) with a 95% confidence interval (CI) was reported for the best-fitted model.

## Results

### Sociodemographic characteristics of study participants

This study included a total of 4167 (weighted) married pregnant women. Nearly two-thirds (65.46%) of study participants had no occupation. About 64 and 42% of pregnant women and their husbands had no formal education respectively. The majority (21.37) of the participants were from households with the richest wealth quantile. Nearly 31% of women had a husband who drinks alcohol and about 47% of study participants were exposed to media. Looking at the community-level characteristics, the majority of participants (82.74%) were rural dwellers and more than half (52%) of them were from agrarian regions. Above three- fourth (80.64%) of the study participants were from communities with high poverty level. About 45% of pregnant women were from communities with high illiteracy level (Table 2).

**Table 2:** The sociodemographic characteristics of study participants

Variables	Weighted Frequency(n)	Percent(%)
Maternal education		
No formal education	2650	63.60
Primary education	1080	25.92
Secondary education	274	6.57
Higher education	163	3.90
Maternal occupation		
Not working	2728	65.46
Working	1439	34.54
Husband education		
No formal education	1744	41.87
Primary education	1307	31.35
Secondary education	820	19.68
Higher education	296	7.10
Husband occupation		
Not working	811	19.47
Working	3356	80.53
Wealth status		
Poorest	806	19.34
Poor	799	19.17
Middle	882	21.18
Rich	790	18.94
Richest	890	21.37
Husband drinks alcohol		
No	2896	69.49
Yes	1271	30.51
Media exposure		
Yes	1536	36.87
No	2631	63.13
Maternal age		
15–24	738	17.70
25–34	1817	43.62
35–39	1231	29.55
40–49	381	9.13
Sex of household head		
Male	3283	78.80
Female	884	21.20
Residence		
Urban	719	17.26
Rural	3447	82.74
Region		
City dewellers	157	3.75
Pastoral	1820	43.68
Agrarian	2190	52.58
Community illiteracy		
Low	2312	55.49
High	1855	44.51
Community poverty level		
Low	807	19.36
High	3360	80.64
Community media exposure		
Low	2217	53.19
High	1950	46.81

### The magnitude of different types of IPV directed to pregnant women

Table 3 presented the weighted magnitude of different forms of intimate partner violence inflicted on pregnant women with 95%CI. Of the variants of IPV, pregnant women were exposed to emotional violence (24%) most frequently. The least prevalent form of IPV was sexual violence. The overall (sexual, physical, or emotional) magnitude of IPV among pregnant women was 28.74 (95% CI **27.38,** 30**.**13).

**Table 3:** The magnitude of different types of IPV directed to pregnant women

Type of IPV	Magnitude	95%CI
Sexual violence	10.21	9.32, 11.17
Physical violence	11.09	10.17,12.08
Emotional violence	24.09	22.82, 25.41
Sexual, physical or emotional	28.74	27.38, 30.13

### Factors associated with intimate partner violence

In the null model the community-level variance [country variance = 0.73; standard error (SE) = 0.10; *P*-value = 0.001], was statistically significant which indicates that there is significant variation in experiencing IPV during pregnancy across communities. This was further supported by the intracluster correlation coefficient which showed that 18.70% of the variation of IPV against pregnant women was attributed to community-level factors.

Moreover, the final model (model IV) indicates that about 45.20% of the variation of IPV directed towards pregnant women is explained by both the individual and community-level factors. The model fitness was assessed by using deviance. Consequently, Model IV was found to be the best-fitted model since it has the lowest deviance value (Table 4).

**Table 4:** Factors associated with IPV directed to pregnant women in Ethiopia

Variables	Model I (null) AOR 95%CI	Model II AOR 95%CI	Model III AOR 95%CI	Model IV AOR 95%CI
Maternal education				
No formal education	–	1.95 (1.16, 3.27)	–	2.07 (1.23, 3.48)**
Primary education	–	1.99 (1.20, 3.28)	–	2.04 (1.24, 3.38)**
Secondary education	–	1.53 (0.90, 2.61)	–	1.53 (1.29.2.62)**
Higher education	–	1.00	–	1.00
Maternal occupation				
Working	–	1.00	–	1.00
Not working	–	0.87 (0.73, 1.03)	–	1.87 (0.74, 1.03)
Husband education				
No formal education		1.26 (0.87, 1.83)	–	1.27 (0.88, 1.84)
Primary education		1.49 (1.04, 2.15)	–	1.47 (1.02, 2.12)*
Secondary education		1.42 (0.98, 2.06)	–	1.36 (0.93, 1.97)
Higher education		1.00	–	1.00
Husband occupation				
Not working		1.08 (0.86, 1.36)	–	1.10 (0.87, 1.39)
Working		1.00	–	1.00
Husband alcohol drinking				
No		2.89 (2.41, 3.45)	–	2.94 (2.36, 3.42)**
Yes		1.00	–	1.00
Wealth status				
Poorest	–	1.28 (0.95, 1.72)	–	1.72 (1.16, 2.56)
Poorer		1.27 (0.93, 1.73)		1.62 (1.09, 2.41)
Middle	–	1.38 (1.02, 1.87)	–	1.74 (1.17, 2.56)
Richer		1.24 (0.92, 1.67)		1.58 (1.08, 2.33)
Richest		1.00		1.00
Media exposure				
No	–	1.12 (0.91,1.37)	–	1.21 (0.98, 1.49)
Yes	–	1.00	–	1.00
Maternal age				
15–24		1.00		1.00
25–34	–	1.15 (0.92, 1.43)	–	1.12 (0.90,1.39)
35–39	–	1.33 (1.05, 1.69)	–	1.28 (1.01, 1.63)*
40–49	–	1.83 (1.33, 2.53)	–	1.78 (1.28, 2.45)**
Sex of household head			–	
Male		1.00	–	1.00
Female		0.93 (0.76, 1.14)	–	0.93 (0.75, 1.14)
Residence				
Urban	–	–	1.00	1.00
Rural	–	–	1.43 (0.96, 2.12)	1.12 (0.71, 1.74)
Region				
City dwellers			1.00	1.00
Pastoral			0.58 (0.41, 0.80)	1.47 (1.04, 1.93)*
Agrarian			0.96 (0.71, 1.29)	1.32 (1.02,1.88)*
Community illiteracy level				
Low	–	–	1.00	1.00
High	–	–	0.96 (0.76,1.20)	1.12 (0.90,1.18)
Community media exposure				
Low	–	–	1.23 (0.98, 1.53)	1.24 (0.98, 1.58)
High	–	–	1.00	1.00
Community poverty level				
Low	–	–	1.00	1.00
High	–	–	0.95 (0.65, 1.39)	0.99 (0.94,1.04)
Random effects				
Community variance (SE)	0.73 (0.10)^*^	0.54 (0.09)^*^	0.52 (0.08)^*^	0.40 (0.07)*
ICC (%)	18.70	14.01	13.80	10.00
PCV (%)	Reference	26.03	28.76	45.20
Model fitness				
Deviance(−2LLR)	4922.08	4707.92	4892.36	4503.22

*Note*: *AOR* Adjusted odds Ratio, *CI* Confidence Interval; * = *P* < 0.05; ** = *P* < 0.01, ICC: intracluster correlation coefficient; PCV: proportional change in variance

Regarding the fixed effects, maternal education, maternal age, wealth index, husband education, husband drinking alcohol, and region were significantly associated with intimate partner violence.

The odds of experiencing intimate partner violence among pregnant women with no education, primary education, and secondary education was 2.07 (AOR = 2.07; 95%CI:1.23, 3.48), 2.04 (AOR = 2.04; 95%CI:1.24, 3.38), and 1.53 (AOR = 1.53; 95%CI:1.29.2.62) respectively times higher as compared to those who had higher education. Besides, pregnant women whose husband has primary education has 47% (AOR = 1.47; 95%CI 1.02, 2.12) increased odds of experiencing IPV as compared to women who had a husband with higher education**.** The likelihood of experiencing IPV among women whose husband drinks alcohol was nearly three folds (AOR = 2.94;95%CI:2.36, 3.42) higher as compared to their counterparts. Regarding wealth index, the odds of experiencing IPV among pregnant women from the poorest, poorer, middle, and richer households was 1.72 (AOR = 1.72; 95%CI: 1.16, 2.56), 1.62 (AOR = 1.62;95% CI:1.09, 2.41), 1.74 (AOR = 1.74;95%CI:1.17, 2.56), and 1.58 (AOR = 1.58;95%CI: 1.08, 2.33) respectively times higher as compared to those from households with richest wealth quantile. The likelihood of experiencing IPV among pregnant women with age category 35–39 and 40–49 was 1.28 (AOR = 1.28;95%CI:1.01, 1.63) and 1.78(AOR = 1.78;95%CI:1.28, 2.45) respectively times higher as compared to women under 15–24 age category. Looking at the region, women from pastoral and agrarian regions had 47% (AOR = 1.47;95%CI:1.04, 1.93) and 32% (AOR = 1.32;95%CI 1.02, 1.88) increased odds of experiencing intimate partner violence respectively as compared to city dwellers (Table 4).

## Discussion

The overall magnitude of intimate partner violence among pregnant women was 28.74 (95% CI 27.38, 30**.**13) with emotional violence at the higher occurrence. Educational status of women and husband, alcohol consumption of husband, age of women, wealth index, and region were significantly associated with experiencing IPV during pregnancy.

The overall magnitude of IPV in the current study was lower than the findings in Nigeria [8], Jordan [42], Kenya [9], Egypt [43], and Portugal [44] and previous Ethiopian studies that were done in Jimma [45] and Tigray [46]. But, it was higher than that of previous studies from Ethiopia that were conducted in the northwest [10] and southern [47] Ethiopia. The lower magnitude of IPV in the current study may be attributed to the differences in culture, social norms, and implementation of laws that prevent violence against women [48]. For example, Ethiopian society is highly patriarchal thus women often feel humiliated and ashamed to disclose violence (most commonly sexual violence) due to fear of negative responses from others within their society because of cultural consequences [49, 50]. Besides, the questions used to assess IPV are culturally sensitive. So, the respondents may not answer such questions honestly. This might lead to underreporting and then low IPV [51, 52]. Furthermore, the difference in gender equality might also contribute to the difference in magnitude of experiencing [53].

This study revealed the higher odds of IPV among women from economically poor households as compared to women from richer households which is supported by the finding in Bangladesh [54]. This might be because women in Ethiopia especially with poor economic status may have minimal access and freedom to utilize the financial resources without consulting their partners which may in turn lead to conflict [55]. In countries like Ethiopia, social responsibility for raising children is vested on women. Therefore, women may be forced to admit the violence from their partner in order not to be separated to minimize the suffering of their children [56].

The odds of experiencing IPV among women with low educational status was higher as compared to those women with secondary and higher education. This finding is consistent with the study in Rwanda [29] and other developing countries [27, 28]. This might be since uneducated pregnant women may have less power to discuss with their partners to minimize any household disputes. It is documented that the likelihood of experiencing violence during pregnancy is negatively affected by low levels of education and lack of decision-making power [57].

Similarly,husband education was also significantly associated with IPV during pregnancy. Women who had a husband with a secondary education level had nearly 50% increased odds of experiencing IPV as compared to those with a husband with higher education. This result is in line with previous studies [9, 27]. It is known fact that education is a source of information and it is a tool to shape a positive behavioral changes. So, uneducated partners may not give freedom to their wives which is commonly driven by cultural beliefs. This study also highlighted another partner-related factor, alcohol consumption. Consistent with the previous findings [9, 26, 29], the likelihood of IPV among women who had a partner that drink alcohol was nearly three folds higher as compared to their counter parts. This might be due to the fact that alcohol facilitates to have violent behaviors [58]. Besides, alcohol use has been associated with having multiple sexual partners, an issue that may also lead to conflict [59].

Age is a significant predictor of IPV during pregnancy. Aged women have higher odds of experiencing IPV as compared to younger ones. This finding is also supported by previous studies in Nigeria [31] and South Africa [32]. This could be because older women might be more likely to report IPV. After all, younger women in Ethiopia are often expected to be submissive, quiet, disciplined, and loyal to their husbands and hence may have a lower probability of reporting IPV.

Similarly, the region was an important predictor of IPV. Women from agrarian and pastoral regions had higher odds of experiencing IPV as compared to city dwellers. The women in urban areas are more autonomous, educated, and well-informed about gender equality. Consequently, they could have confidence in decision-making in the household [60].

The strengths of this study were; first, it was conducted using data from a large national survey which provides adequate power to detect the true effect of the independent variables. Second, the sampling weight was applied during the analysis to get reliable estimates and standard errors. As a limitation, since the study used cross-sectional data, a causal relationship between IPV and the identified independent variables cannot be established.

## Conclusion

Intimate partner violence during pregnancy is a public health problem in Ethiopia. The educational status of women and their husbands, wealth index, age of women, alcohol consumption of husband, and region were significantly associated with intimate partner violence during pregnancy. Therefore, improving the educational status of women and their husbands, improving the economic capacity of women, and promoting the healthy behavior of husbands by reducing alcohol consumption is vital to reduce the magnitude of IPV and its consequences in Ethiopia. Besides, minimizing dominant patriarchal ideologies which privilege heterosexual marriage through cultural and religious connections could also play a central role in reducing IPV. In general, the gender issue is prioritized as an essential aspect in accelerating the united nation’s 2030 global agenda [61]. Ethiopia had also considered gender equality as a transformative policy [62] Therefore, the findings in this study could have a positive effect towards achieving the SDG goals in Ethiopia as it provides a piece of evidence for policymakers and program designers to make an informed decision.

## Data Availability

The datasets we used for this study were publicly available at http://www.dhsprogram.com. Website.

## Abbreviations

CSA: Central Statistical Agency
DHS: Demographic and health survey
EAs: Enumeration Areas
EDHS: Ethiopian Demographic and Health Survey
IPV: Intimate partner violence
WHO: World Health Organization
